# Executive function predicts risk of falls in older adults without balance impairment

**DOI:** 10.1186/1471-2318-11-74

**Published:** 2011-11-09

**Authors:** Teresa J Buracchio, Nora C Mattek, Hiroko H Dodge, Tamara L Hayes, Misha Pavel, Diane B Howieson, Jeffrey A Kaye

**Affiliations:** 1 Department of Neurology, Oregon Health & Science University, 3181 SW Sam Jackson Park Road, CR-131, Portland, OR 97239 USA; 2 Neurology Service, Portland Veterans Affairs Medical Center, 3710 SW U.S. Veterans Hospital Road, Portland, OR 97239 USA; 3 Oregon Center for Aging & Technology, Oregon Health & Science University, 3181 SW Sam Jackson Park Road, Portland, OR 97239 USA; 4 Department of Biomedical Engineering, Oregon Health & Science University, 3303 SW Bond Avenue, Portland, OR 97239 USA

## Abstract

**Background:**

Executive dysfunction has previously been found to be a risk factor for falls. The aim of this study is to investigate the association between executive dysfunction and risk of falling and to determine if this association is independent of balance.

**Methods:**

Participants were 188 community-dwelling individuals aged 65 and older. All participants underwent baseline and annual evaluations with review of health history, standardized neurologic examination, neuropsychological testing, and qualitative and quantitative assessment of motor function. Falls were recorded prospectively using weekly online health forms.

**Results:**

During 13 months of follow-up, there were 65 of 188 participants (34.6%) who reported at least one fall. Univariate analysis showed that fallers were more likely to have lower baseline scores in executive function than non-fallers (p = 0.03). Among participants without balance impairment we found that higher executive function z-scores were associated with lower fall counts (p = 0.03) after adjustment for age, sex, health status and prior history of falls using negative binomial regression models. This relationship was not present among participants with poor balance.

**Conclusions:**

Lower scores on executive function tests are a risk factor for falls in participants with minimal balance impairment. However, this effect is attenuated in individuals with poor balance where physical or more direct motor systems factors may play a greater role in fall risk.

## Background

Falls are common in older persons with approximately one third of individuals aged 65 and older experiencing a fall annually [1]. Falls are the leading cause of fatal and non-fatal injuries in this population and are a significant public health concern [2]. Identification of potentially modifiable risk factors is important for developing fall prevention strategies and interventions. Previously identified risk factors for falls include a number of physical impairments such as impairment of gait and balance, vision impairment, orthostatic hypotension, musculoskeletal problems, and also the use of some medications [1,3-9]

The presence of cognitive impairment has also been identified as a risk factor for falls [10-12]. Lower scores on cognitive screening tests such as the Mini-Mental State Examination and the Montreal Cognitive Assessment have been associated with an increased risk of falls [11,12]. Several studies have examined the role of specific cognitive domains on fall risk. Lower scores on tests of attention, executive function, memory and visuospatial function have all been reported to be associated with an increased risk of falls in both cognitively intact and cognitively impaired individuals [13-16]. Difficulty with dual-task walking, a measure of divided attention and executive function in which individuals are given a secondary mental task while walking, has consistently been shown to be associated with an increased risk of falls [17-19]

Although most studies of cognitive function and falls include gait assessments in their study design, balance is often measured only indirectly through gait assessments and specific balance functions may not be included in the analyses [13,15,16]. Given the limited information available about the association of balance with cognitive function and falling, we wished to examine the role of balance in the association between cognitive dysfunction, and specifically executive dysfunction, and falling. In this study, we wished to answer the following questions: Which cognitive domains are associated with an increased risk of falling, and specifically, is executive function associated with an increased risk of falling? Is the association between executive function and falling independent of balance? We hypothesized that the association between executive dysfunction and falls would be independent of balance impairment. In order to answer these questions, we performed a secondary analysis of existing data from two longitudinal cohort studies using baseline neuropsychological and motor testing and prospective measurement of falls over a 13 month period.

## Methods

This study is a secondary analysis of existing data from two longitudinal cohort studies developed by the Oregon Center for Aging and Technology (ORCATECH) at Oregon Health & Science University (OHSU): the ORCATECH Living Laboratory study and the Intelligent Systems for Assessing Aging Change (ISAAC) study. Both studies use the same in-home sensor technology and computers to assess behavioral and cognitive changes that occur with aging. The ORCATECH Living Laboratory study cohort was originally a pilot study for ISAAC. Study procedures were identical for the two studies and have been previously described [20]

### Participants

Briefly, participants were recruited from local senior centers and retirement communities in the Portland, Oregon metropolitan area and from other OHSU Layton Aging & Alzheimer's Disease Center studies. Inclusion criteria were being 60 years and older for the Living Laboratory study and 80 years and older (or 70 years and older for minorities and those living with participants 80 and older) for the ISAAC study, living independently without a formal caregiver, not demented with a Clinical Dementia Rating Score ≤ 0.5, [21]. and being of average health for age with either no or well-controlled chronic health conditions. Participants with an MMSE ≤24 or a CDR of 1 and met eligibility criteria were enrolled if they lived with a spouse or partner who was participating in the study. Exclusion criteria were health conditions that may limit physical participation or lead to death within 3 years. The study protocols were approved by the OHSU Institutional Review Board (Living Laboratory IRB# 2765; ISAAC IRB# 2353).

Between November 2006 and September 2009, there were 36 participants who signed informed consents and were enrolled in the Living Laboratory. All Living Laboratory participants underwent baseline evaluations and had computers installed in their home. Between March 2007 and September 2009, 265 participants were initially screened for the ISAAC study and signed informed consents. Of these, 32 participants either did not meet inclusion criteria or lived with another resident who did not wish to participate. The remaining 233 participants met inclusion criteria and underwent baseline evaluations and had computers installed in their home. Since the initial enrollment, there have been 18 participants who withdrew from the study protocol and 14 deaths.

In August 2009, both studies began collecting detailed prospective information about falls using weekly online health forms. This study focuses on the subset of 201 participants who participated in these weekly online health forms between August 23, 2009 and September 22, 2010. Because we wished to focus on independent, ambulatory, non-demented elderly, we excluded individuals at a high risk for falls due to physical impairments or with significant cognitive impairment. There were 13 participants excluded from the analysis: Parkinson's disease (n = 1), significant vision impairment that impaired daily functioning (n = 3), wheelchair bound individuals (n = 1), MMSE < 24 (n = 7) and CDR of 1 (n = 1). Therefore, there were 188 participants included in this analysis. The participants who were excluded from the analysis were more likely to be men (p = 0.03) than those included in the analysis but were similar in age (p = 0.24) and years of education (p = 0.11).

### Clinical Assessment

Participants underwent baseline and annual in-home clinical evaluations by research clinicians that included a review of medical histories and standardized physical and neurologic examinations. Medical histories and medication lists were reviewed at each visit. Health status was documented with the modified Cumulative Illness Rating Scale (CIRS) [22]. The musculoskeletal subscore of the CIRS was used to assess for the presence of arthralgias and myalgias that may affect mobility. Medication lists were reviewed for central nervous system-active medications such as sedatives, antidepressants, antipsychotics and antiepileptic drugs since these have been shown to be associated with fall risk and the total number was recorded [4,23]. The Geriatric Depression Scale was used to screen for depressive symptoms [24]

### Motor testing

In addition to a standardized neurologic examination that included assessment of motor function, additional quantitative and qualitative measures of motor function were obtained. Gait speed was assessed by asking participants to walk from a starting point to a marker 15 feet away, turn, and back at a normal casual gait for a total of 30 feet (9.14 meters) [25]. Time in seconds was measured with a stopwatch to the nearest second for two trials and the mean recorded. The motor portion of the Unified Parkinson's Disease Rating Scale (UPDRS) was administered [26]. Balance and gait were assessed using the Performance-Oriented Assessment of Mobility described by Tinetti [27]. The balance scale measures balance sitting, rising and standing and with challenging tasks such as bending forward and turning. The gait scale measures elements of the participant's usual casual gait such as step length and symmetry. There have been many different scoring systems reported for this scale [28]. By convention at our center, balance is measured on a scale of 0-26 and gait is measured on a scale of 0-9 with higher scores indicating better performance or fewer deficits.

### Cognitive testing

Participants underwent baseline and annual standardized neuropsychological testing that included the National Alzheimer's Coordinating Center (NACC) protocol [29]. The MMSE and Cognistat [30]. were performed as brief cognitive evaluations. Because many of the neuropsychological test scores are not normally distributed and to avoid misrepresentation of the cognitive function of the participants by poor performance on any single test, a z-score was created for each cognitive domain tabulated from 2-3 representative neuropsychological tests for each domain. Cognitive domain z-scores were calculated using the group mean and standard deviation for the raw scores of each neuropsychological test from a normative sample including all cognitively intact participants (CDR = 0) at baseline. The scores were z-normalized, summed, and averaged for each cognitive domain. An overall global score was derived from all 13 tests. Executive function was assessed with the Trail Making Test-Part B and Category Fluency Animals and Vegetables [31]. Working memory was assessed with Letter-Number Sequencing (WMS-III) [32]. and Digit Span Backward (WAIS-R) [33]. Attention/processing speed was assessed with Digit Span Forward, [34] the Digit-Symbol Test, [34] and Trail Making Part A. Memory was assessed with Logical Memory Delayed (WMS-R), [33]. Visual Reproduction II, [33] and the CERAD Word-List Recall [35]. Visuospatial function was assessed with Block Design (WAIS-R) [33] and Picture Completion (WAIS-R) [33]

### Falls

Incident falls were prospectively reported weekly on a computer provided by the study with online health forms. Once a week, participants received a computerized form when they logged in to their study-provided computer terminal that inquired about health events, medication changes, or changes in their living situation. A question on the form asked participants how many times they had fallen in the prior week. Falls were defined as any fall, including a slip or trip, in which the subject came to rest on the floor, ground or on a lower level [36]. Fallers were defined as participants who had one or more falls during the 13-month follow-up period. Participants who did not fill out a form for two consecutive weeks were contacted by a research assistant by phone and the falls information for that two week period was recorded. All participants included in this study continuously participated with the online health forms during the follow-up period and there was no attrition to computer use. Number of falls in the prior year was obtained with the Oregon Gait and Balance Inventory at the baseline evaluation [17]

## Statistical Analysis

Subject characteristics and cognitive data were obtained from the most recent clinical evaluation within one year preceding the start of the falls forms on August 23, 2009. Characteristics were compared between non-fallers and fallers using Student's t-test or Wilcoxon Ranked Sum Test for continuous variables and Pearson Chi-Square test for categorical variables.

Negative binomial regression models were used to investigate the relationships between each cognitive domain z-score as well as the global z-score (independent variables) at baseline and number of falls (outcome variable) reported during the 13 months of follow-up. Models included variables that were found to be significantly different between the two groups on univariate analysis (history of prior falls, modified CIRS, and Tinetti balance score). Age and sex were included in all models due to their known association with risk of falls [1,3,37]. Because we wished to examine the role of cognitive function on the risk of falling relative to balance, we performed additional analyses using negative binomial regression to investigate the relationships between each cognitive domain z-score and number of falls among individuals with and without balance impairment. Balance impairment was defined as a score < 24 (median among the entire cohort) of maximum score 26 on the Tinetti balance scale. Model goodness of fit was assessed using the ratio of the Deviance measure to DF (degrees of freedom) [38]. Analyses were carried out using SAS 9.2 (SAS Institute Inc., Cary, NC).

## Results

The study cohort of 188 participants had a mean age of 83.2 (SD 6.6) years and 140 (74.1%) were women. Participants were generally healthy with a mean CIRS score of 21.3 (SD 2.9) (modified CIRS scores range from 14 to 70; lower scores indicate less illness). There were 65 (34.6%) participants who reported at least one fall during the follow-up period. Twenty-four participants reported more than one fall and one subject reported ten falls. Fallers were more likely to have a lower CIRS score (p = 0.03), perform worse on the Tinetti balance scale (p < 0.01), and report a prior history of falls (p = 0.01) than non-fallers. Demographic and health characteristics of fallers and non-fallers are summarized in Table 1. Neuropsychological test score summary measures for fallers and non-fallers are presented in Table 2.

**Table 1:** Clinical and demographic characteristics of fallers and non-fallers

Variables	Non-Faller (n = 123)	Faller (n = 65)	p value
Age	82.8 (7.0)	83.9 (5.7)	0.25

Sex (#, % women)*	91 (73.4%)	49 (75.4%)	0.77

Education (yrs)	15.2 (2.9)	15.1 (2.7)	0.83

MMSE**	28.3 (1.7)	28.8 (1.3)	0.12

CIRS (standard test range 14-70)**	21.0 (2.9)	21.9 (2.8)	0.03

CIRS musculoskeletal subscore (standard test range1-5) **	2.2 (0.6)	2.3 (0.5)	0.42

Geriatric Depression Scale**	1.3 (2.0)	1.4 (1.8)	0.52

CNS medications**	0.3 (0.7)	0.3 (0.6)	0.56

Tinetti Balance** (max score: 26)	22.8 (4.5)	21.2 (4.9)	< 0.01

Tinetti Gait** (max score: 9)	7.9 (1.8)	7.6 (2.0)	0.29

UPDRS**	0.5 (1.1)	0.7 (1.2)	0.18

History of previous falls (#, %)*	23 (19.5%)	23 (36.5%)	0.01

Gait speed (cm/s)	80.8 (21.2)	76.5 (25.3)	0.21

*Pearson Chi-square test **Wilcoxon rank-sum test

**Table 2:** Neuropsychological test scores of fallers and non-fallers

Variables	Non-Faller (n = 123)	Faller (n = 65)
*Executive Function domain*		

Category Fluency: Animals	17.8 (5.1)	16.9 (5.2)

Category Fluency: Vegetables	13.9 (4.2)	12.3 (3.9)

Trail Making Part B	112.8 (53.9)	126.4 (66.2)

*Working Memory domain*		

Digit Span Backward	4.5 (1.2)	4.6 (1.2)

Letter-Number Sequencing	7.8 (2.3)	8.2 (2.5)

*Attention/Processing Speed domain*		

Digit Span Forward	6.7 (1.0)	6.8 (0.9)

Digit-Symbol Test	39.0 (11.5)	38.3 (9.1)

Trail Making Part A	41.6 (19.0)	45.1 (21.9)

*Memory domain*		

Logical Memory Delayed	11.5 (4.6)	11.9 (4.4)

Visual Reproduction II	18.6 (10.5)	19.2 (10.0)

CERAD Word List Recall	6.5 (2.5)	6.8 (2.0)

*Visuospatial domain*		

Block Design	21.3 (8.2)	21.8 (7.2)

Picture Completion	13.0 (3.7)	13.3 (3.5)

To determine the underlying cognitive factors that may account for an increased likelihood of falling, univariate analyses compared global and domain-specific cognitive z-scores between fallers and non-fallers. There was no association between the global cognition z-score and likelihood of falling and within individual cognitive domains, only the executive function z-score was significantly lower in fallers than in non-fallers (-0.19 vs 0.08, p = 0.03). When adjusted for age, sex, CIRS, prior falls and Tinetti balance in a negative binomial regression model, higher executive function z-score remained associated with lower fall counts at p = 0.10 (Table 3, Model 1).

**Table 3:** Negative binomial regression models showing relationship between executive function, falling and balance

	Model 1 Full Cohort *n = 188*		Model 2 No Balance Impairment Subset *n = 96*		Model 3 Balance Impairment Subset *n = 92*	
**Covariate**	**Coefficient**	**p-value**	**Coefficient**	**p-value**	**Coefficient**	**p-value**

Executive function z-score	-0.30	< 0.10	-0.56	0.03*	-0.18	0.41

Age (yrs)	-0.01	0.58	0.06	0.08	-0.05	0.04*

Female	-0.03	0.91	-0.36	0.43	0.10	0.79

Cumulative Illness Rating scale	0.06	0.25	0.18	0.04*	0.01	0.82

Prior fall history	0.83	0.004**	0.49	0.28	0.84	0.01*

Tinetti Balance	-0.02	0.52				

*: p < 0.05, **: p < 0.01

### Balance impairment

We further assessed the association between executive function and number of falls among those with no or minimal balance impairment (Tinetti balance scale > 23 of 26, n = 96) and those with balance impairment (Tinetti balance scale < = 23, n = 92) separately. We chose this cut-point based off of the cohort median Tinetti balance score of 24. Twenty-seven (28%) of participants with no balance impairment reported prospective falls while thirty-nine (42%) of participants with balance impairment reported falls during the study period.

For those with no or minimal balance impairment, we found that higher executive function z-scores were associated with lower fall counts when adjusted for age, sex, CIRS score and prior falls (p = 0.03) (Table 3, Model 2) such that, for a one standard deviation increase in executive function z-score, the difference in the log of expected fall counts is expected to change by -0.56, given the other predictor variables in the model are held constant. Interestingly, prior history of falls was not a significant predictor of prospective falls in our cohort of older adults with no balance impairment (p = 0.28).

For individuals with balance impairment, the relationship between executive function z-score and risk of falls was non-significant after adjustment for the same covariates (Table 3, Model 3). A prior history of falls was a significant predictor of future falls only in our older adults with balance impairment (0.01).

## Discussion

In this cohort of community-dwelling non-demented older adults, we found that fallers are more likely to have lower scores in executive function. However, this finding is attenuated in individuals with poor balance as measured by the Tinetti balance scale. These executive function scores, although lower, were not in a range that would suggest dementia or mild cognitive impairment. Thus in independently functioning, non-demented older persons, the degree to which executive function is associated with the risk for falling appears to be dominated by the degree of balance impairment. This finding was contrary to our hypothesis that participants with lower executive function scores would be at a greater risk of incident falls regardless of the presence of balance impairment. It should also be noted that the mean gait velocity of both fallers and non-fallers was less than 1 m/s which places our cohort at a high risk of falls at baseline [39]

We suggest that basic physical function and ultimately poor balance overwhelm the effect of relatively minor changes in executive function with aging with respect to remaining upright when experiencing a falling event. This does not mean that executive dysfunction is not important, but suggests that in those with additional balance impairment, these changes in cognitive function may play a lesser role and thus imply that fall interventions need to take into account both the cognitive and balance domains.

Our findings are consistent with prior studies that have found impairment of executive function as a risk factor for falls in cognitively intact individuals [13,14]. However, in contrast to our study, these studies did not explore the specific role of balance in this association. Holtzer et al. included assessments of gait impairment in their analysis which may include elements of balance, but they did not include specific measurements of balance [13]. Herman et al. included balance assessments in their study, but limited their cohort to individuals without a prior history of falls likely excluding those with more severe balance impairments [14]

The association between cognitive function and balance as measured independently from gait has not been fully explored in older adults. Balance and gait are closely related and balance is often measured only indirectly through gait assessments in studies of cognition and fall risk [13,40]. Studies that examine cognitive status using formal cognitive testing and isolated measures of balance are lacking in healthy older adult. However, some studies suggest that there is an association between balance and executive function that is independent of gait performance. A study that measured balance in older adults using force plates found that the performance of cognitively-demanding attentional tasks increased postural sway, particularly in subjects with a prior history of falls [41]. Another study that examined older subjects with a prior history of mild stroke found that better performance on the Stroop Test was positively correlated with better performance on the Berg Balance Scale [42]

Attention and executive function appear to play an important role in the higher order cognitive control of gait, posture, and balance. Individuals with deficits in these areas of cognition have been shown to have slower gait velocity, greater gait variability, and perform more poorly on tasks of stepping function [43,44,40]. Deficits in attention and executive function may lead to a decompensation of higher-order gait and postural control and increase the risk for falls. It remains unclear whether these declines in cognitive function are part of the normal aging process or related to underlying pathology. Prior neuroimaging studies have shown that white matter changes, particularly in the deep frontal lobes, are associated with increased risk of falls [45]. As both motor and executive functions are prominently controlled by frontal systems, this may account to some extent for both the cognitive and gait changes that are risk factors for falls.

The use of cognitive training interventions that focus on improving executive function by developing skills such as dual tasking is an active area of investigation for fall prevention [46,47]. Our data suggests how such therapies could be tailored to the individual. Those without significant balance problems may benefit from cognitive training for fall prevention. However, those with prominent balance problems may not be amenable to cognitive interventions and may be best served by emphasis on other fall prevention therapies such as strength and balance training. Further clinical trials are needed to determine if these methods are effective for fall prevention.

There were several strengths to our study. We used a longitudinal study design with prospective measurement of falls that strengthened our ability to find associations. The major strength of our study was the prospective collection of the falls date on a weekly basis that minimizes recall bias [48]. Our study population was a generally healthy, community-based cohort. We used standardized, validated measurements of motor function and cognitive function and a standardized definition of falls [36]

There are limitations to our study. Although this study collects information of falls it was not initially designed as a study to assess falls risk factors, therefore some known risk factors for falls such as orthostatic hypotension were not assessed and cannot be included in the analyses. We collected the data using a novel data collection technique of online health forms that has not been validated. However, this appears to be a valid measure of falls as our fall rate of 35% is comparable to the incidence reported in prior falls studies using prospective measurement with diaries [1,3,37]. As our cohort is selected for their interest and willingness to participate in a study using technology, it is not clear if computerized falls forms would be acceptable to the general population of older adults. Our cohort is also unique in that they are older, predominantly women, well-educated and have agreed to in-home monitoring. This may limit the ability to generalize our findings to the general population of older adults.

## Conclusions

In this study, we performed a secondary analysis of an existing dataset to examine the association of baseline executive function with the likelihood of falling using a prospective measurement of falls and standardized neuropsychological and motor testing. We found lower baseline scores on executive function measures were associated with an increased likelihood of falling. We performed additional analyses to examine if this association is independent of balance. When divided into groups for presence of balance impairment, we found that higher executive function z-scores were associated with lower fall counts in those with minimal balance impairment (p = 0.03) when adjusted for age, sex, CIRS and prior falls but was non-significant in those with balance impairment. While lower scores on executive function tests are a risk factor for falls in participants with minimal balance impairment, this effect is attenuated in individuals with poor balance where physical or more direct motor systems factors may play a greater role in fall risk.

## Competing interests

Dr. Hayes has a significant financial interest in Intel. There are no other conflicts of interest to report.

## Authors' contributions

Study concept and design: TB, NM, JK; Acquisition of data: JK, TH; Statistical analysis: TB, NM, HD; Interpretation of data: TB, NM, HD, JK, DH; Drafting of the manuscript: TB; Critical revision of the manuscript: TB, NM, HD, TH, MP, DH, JK. All authors read and approved the final manuscript.

## Pre-publication history

The pre-publication history for this paper can be accessed here:

http://www.biomedcentral.com/1471-2318/11/74/prepub
